# A Study of Misconceptions About Appendicitis Among the Resident Population of the Aseer Region

**DOI:** 10.7759/cureus.45229

**Published:** 2023-09-14

**Authors:** Fahad S Al Amri, Reem T Alalyani, Renad M Alshehri, Yousef T Alalyani, Lubna M Ladnah, Tariq M Ladnah, Alhanouf Alqahtani

**Affiliations:** 1 Department of Surgery, College of Medicine, King Khalid University, Abha, SAU; 2 Department of Medicine and Surgery, College of Medicine, King Khalid University, Abha, SAU; 3 General Practice, Al Madha General Hospital, Khamis Mushait, SAU; 4 General Practice, Alfirsha General Hospital, Khamis Mushait, SAU

**Keywords:** awareness, healthcare knowledge, aseer region, misconceptions, appendicitis

## Abstract

Background: Appendicitis is a common abdominal emergency requiring swift medical intervention. Misconceptions about this condition can lead to delayed diagnosis and potentially life-threatening complications. In the Aseer region of Saudi Arabia, where healthcare accessibility and awareness levels vary, addressing such misconceptions is of paramount importance. The aim of this study is to investigate and identify the prevalent misconceptions regarding appendicitis among the resident population of the Aseer region. Understanding the prevalent misconceptions and knowledge gaps is essential to develop targeted educational interventions and enhance public awareness.

Methods: This study utilized a cross-sectional study design to investigate misconceptions about appendicitis among residents in the Aseer region. Over a period of three months, 329 Aseer region resident population were interviewed. The symptoms, causes, diagnosis, treatment, and preventive measures of appendicitis were all covered in a questionnaire that was created to gather information on people's knowledge of appendicitis. Data were collected using an online questionnaire. Descriptive analysis was performed using frequencies and percentages, while inferential analysis employed appropriate statistical tests such as chi-square.

Results: The study's 329 participants were made up of 56% men and 44% women. 40% of the sample size was between the ages of 18 and 30, 26% were between the ages of 31 and 40, 15% were between the ages of 41 and 50, and 10% were above 50 years, with those under the age of 18 years accounting for the smallest proportion (9%). The majority of the respondents (37%) were college graduates, 25% were college students, 23% were in high school and 15% were in middle school. Chi-square tests were conducted to examine the associations between background knowledge and pain area, as well as between background knowledge and source of information. For the association between background knowledge and pain area, the Chi-square test yielded a significant result (X² = 9.104, p = 0.028); the Chi-square test also revealed a significant result (X² = 8.078, p = 0.044) between background knowledge and the source of information about appendicitis.

Conclusion: The analysis suggests a notable knowledge gap among the participants, with a significant portion displaying limited understanding or responding with "I don't know" when queried about appendicitis. It is important to note that this observation includes middle school students, who may be too young to be expected to possess knowledge about medical conditions. Additionally, there appears to be gender-related variation in opinions, misconceptions, and understanding regarding appendicitis.

## Introduction

Appendicitis is a common medical condition characterized by inflammation of the appendix. It is a potentially serious condition that requires prompt medical attention, if untreated appendicitis can lead to complications such as rupture and peritonitis. A diversified population with variable access to healthcare and education can be found in the Aseer region in southwest Saudi Arabia. Understanding the prevalent misconceptions and knowledge gaps is essential to develop targeted educational interventions and enhance public awareness.

Although generally located in the right lower quadrant of the abdomen, the appendix may rarely be found in any part of the abdomen depending on whether or not there were any aberrant developmental concerns, such as malrotation of the midgut, or if there are any other particular situations, such as pregnancy or previous abdominal surgeries. It is important to note that the symptoms of this disorder are similar to those of a hernia. During the fifth week of pregnancy, the embryonic stage of appendix development begins. There is a rotation of the midgut towards the external umbilical cord, followed by a return to the abdominal cavity and another rotation of the cecum. As a consequence, the appendix is then located in its customary retrocecal position. It is frequently a sickness that manifests itself suddenly, typically within 24 hours, but it is also capable of masquerading as a condition that is more persistent. In cases when there has been a perforation but the abscess has been controlled, the symptoms may be less severe. The precise role that the appendix plays in the body has been the subject of much discussion. Particularly in younger people, it is generally acknowledged that this organ performs the function of a lymphoid organ in addition to perhaps having an immunoprotective role. According to some hypotheses, the appendix serves as a repository for "healthy" bacteria that are found in the colon [1- 4]. Still, there are many who maintain that it is little more than an evolutionary relic and serves no practical use.

Although acute appendicitis is frequent, it is subject to common misconceptions [5]. Since time immemorial, appendicitis has been understood to be a progressive inflammatory condition that can be most effectively managed by performing an appendectomy as soon as possible. According to the findings of a recent meta-analysis that compared initial treatment with antibiotics alone and appendectomy, antibiotic therapy may be a viable alternative to appendectomy for certain patients [6]. However, there is a paucity of information regarding patients' understanding of appendicitis, the outcomes they prioritise, and their treatment preferences [6]. For an early diagnosis and the best course of treatment, it is essential to accurately and promptly recognise the signs and symptoms of appendicitis. However, misunderstandings and ignorance about appendicitis among the general public might obstruct accurate comprehension of the condition, resulting in postponed treatment and higher morbidity.

The purpose of this study is to investigate and identify the common misconceptions regarding appendicitis in order to raise public awareness and promote knowledge about this condition among residents of the Aseer region. There is a severe lack of studies on the inhabitants' false beliefs regarding appendicitis in the particular setting of the Aseer region. Knowing the common misunderstandings and information gaps concerning appendicitis is essential for creating effective educational interventions, especially given the distinctive cultural and demographic characteristics of the Aseer region.

## Materials and methods

Study design and settings

The study used a cross-sectional survey approach. We utilized a non-probability sampling method which was conducted among residents of the Aseer region of Saudi Arabia. A total of 329 Aseer region resident population were interviewed. For data collection, we used a questionnaire that has been previously validated in other publications and conducted on a population similar to the population in our study. We made slight modifications to ensure alignment with our studied population. The questionnaire was converted to electronic means using Google Forms. Data were collected using an online questionnaire. The inclusion criteria include residents of the Aseer region of Saudi Arabia, while the exclusion criteria include nonresidents of the Aseer region of Saudi Arabia. The study population was the general population and the duration of the study was three months.

Sampling technique

Non-probability sampling method, i.e., convenience sampling was adopted for this study. The participants were chosen based on their availability and willingness to participate in the study.

Ethical consideration

The study was conducted in accordance with the Research Ethics Committee at King Khalid University (HAPO-06-B-001) approved on 30 May 2023 with approval number ECM#2023-2113.

Statistical analysis

The collected data was cleaned and visualized using Microsoft Excel (2016). Statistical analysis was performed using Statistical Package for Social Sciences (SPSS) version 22 software (IBM Corp., Armonk, NY). Categorical variables were represented using descriptive statistics, including total numbers and percentages. Comparison between variables was performed using a Chi-square (X2) test. The prevalence was given in percentage with a 95% confidence level. A test with a p-value < 0.05 was considered statistically significant.

## Results

Table 1 provides valuable insights into the demographic characteristics, educational background, and knowledge regarding the function of the appendix among the respondents in the study. From the data, it was observed that the majority of the respondents were in the age group of 18-30 years 132(40%), and the participants included both males 184(56%) and females 145(44%). In terms of educational qualification, the largest proportion of respondents were college graduates 122(37%), followed by college students 82(25%).

**Table 1:** Sociodemographic Characteristics of the Respondents N=number of respondents; %=percentage

		N	%
Age (years)	<18	29	9
	18-30	132	40
	31-40	86	26
	41-50	49	15
	>50	33	10
Total		329	100
Gender	Male	184	56
	Female	145	44
Total		329	100
Educational Qualification	College Graduate	122	37
	College student	82	25
	High school	49	15
	Middle School	76	23
Total		329	100

Table 2 presents the respondents' answers to the background knowledge, source of information, and pain area in appendicitis. The majority of participants who were asked about their prior knowledge indicated that they responded "I don't know" 100(30.4%) while 89(27%), 72(21.9%), and 68(20.7%) responded having medium, excellent and good levels of background knowledge, respectively. This indicates that a sizable percentage of participants knew little to nothing about appendicitis. About 92(28%) of the respondents reported that their source of information with respect to appendicitis is hospital through the doctors, 89(27.1%) reported that their source of information was family members, 72(21.9%) and 76(23%) revealed that their source of information was through social media and Google respectively. When asked about their own pain experiences, the majority of participants, 100 (30.4%), reported discomfort in their stomach region, while 65 (19.8%) and 91 (27.7%) reported feeling pain in their left upper abdomen and right lower abdomen, respectively. Approximately 73 (22.1%) participants were unsure of the exact location of their pain.

**Table 2:** Background Knowledge, Source of Information, and Pain Area in Appendicitis N=number of respondents; %=percentage

		N	%
Background Knowledge	Excellent	72	21.9
	Good	68	20.7
	Medium	89	27
	I don't know	100	30.4
Total		329	100
Source of Information	Doctor	92	28
	Family member	89	27.1
	Social Media	72	21.9
	Google	76	23
Total		329	100
Pain Area	Stomach	100	30.4
	Left Upper Abdomen	65	19.8
	Right Lower Abdomen	91	27.7
	I don't Know	73	22.1
Total		329	100

The analysis conducted to evaluate the relationship between background knowledge and pain area indicates that there is a meaningful and statistically significant association between these two variables (X² = 9.104, p = 0.028). Additionally, the results suggest that there exists a weaker but still noticeable association between background knowledge and the source of information (X² = 8.078, p = 0.044). These findings suggest that background knowledge plays a role in understanding the pain experienced and may also have some influence on the information sources sought in cases of appendicitis (Table 3).

**Table 3:** Association between Background Knowledge, Source of Information, and Pain Area in Appendicitis Chi-square (X2), significant at p<0.05

Variables	X²	P-value
Background Knowledge vs Pain Area	9.104	0.028
Background Knowledge vs Source of Information	8.078	0.044

Table 4 presents the assessment of the respondents' knowledge about symptoms and treatments related to appendicitis. the majority of respondents were aware of common symptoms such as nausea and vomiting (29.1%), muscle cramps (14.9%), and numbness of extremities (23%). However, a notable portion responded with "I don't know" (28%), indicating a lack of knowledge about appendicitis symptoms among some participants. Regarding treatment options, the most recognized treatments in emergency cases of acute appendicitis were surgery (39%) and antibiotics (45%). A smaller proportion responded with "None" (17%). When asked about other treatments besides surgery and antibiotics, the responses varied. The most commonly mentioned alternatives were herbs (22%), garlic (18%), and ginger (20%). Some respondents also mentioned yogurt and milk (12%) and warm compressors (6%) as possible treatments.

**Table 4:** Assessment of the Knowledge of the Respondents on the Symptoms and Treatments in Appendicitis N=number of respondents; %=percentage

		N	%
Symptoms	Muscle cramps	49	14.9
	Nausea and Vomiting	96	29.1
	Rash	17	5
	Numbness of Extremities	76	23
	I don't know	91	28
Total		329	100
Treatment in emergency cases of acute appendicitis	Surgery	125	39
	Antibiotics	148	45
	None	56	17
Total		329	100
Is there treatment other than antibiotics and surgery?	Yes	257	78
	No	72	22
Total		329	100
What treatment do you know other than surgery and antibiotics?	Herbs	72	22
	Garlic	59	18
	Ginger	66	20
	Yoghurt and milk	40	12
	Warm compressor	20	6
Total		257	78

Table 5 presents the results of a Chi-square test analyzing the association between gender and respondents' answers to three questions. The responses regarding previous appendicitis surgery and perceived risks of surgical removal showed statistically significant associations with gender (p<0.05). However, there was no significant difference between genders regarding their views on surgery as the best treatment for appendicitis (X2=0.676, p>0.05).

**Table 5:** Respondents' Opinions on the use of Surgery for the Treatment Among Genders in the study X2=chi-square; df=degree of freedom; p-value significant at >0.05 Study participants: male =184, female =145

		Male N(%)	Female N(%)	X^2 ^(df)	P-value
Have you undergone surgery for appendicitis before?	Yes	84(45.7%)	60(41.4%)	4.59(1)	<0.05
	No	100(54.3%)	85(58.6%)		
Do you think surgery is the best treatment for appendicitis?	Yes	67(36.4%)	58(40.0%)	0.676(1)	>0.05
	No	117(63.6%)	87(60.0%)		
What are the risks of surgical removal of the appendix?	Hernia	32(17.4%)	18(12.4%)	8.47(1)	<0.05
	Obesity	60(32.6%)	40(27.6%)		
	Loss of important functional organ	52(28.3%)	27(18.6%)		
	I don't know	40(21.7%)	60(41.4%)		

## Discussion

Acute appendicitis (AA) is a common cause of lower abdominal pain that prompts patients to seek emergency medical attention and is the most prevalent diagnosis among young individuals admitted with an acute abdomen [7]. The data presented in Tables 1 to 5 offer valuable insights into the demographics, knowledge, and attitudes of respondents regarding appendicitis in the study area. The findings of this research shed light on crucial aspects of respondents' characteristics and their understanding of the signs, symptoms, treatments, and risks associated with appendicitis.

Regarding demographics, the majority of study participants fell within the age group of 18 to 30 years, aligning with existing research that indicates appendicitis predominantly affects young individuals [7-9]. The balanced representation of men and women in the study enabled a comprehensive examination of gender-related knowledge and attitude disparities.

Concerning findings related to respondents' background knowledge and sources of information. A significant proportion of participants reported limited knowledge about appendicitis, with many responding with "I don't know." These findings are consistent with previous studies that have identified knowledge gaps among the general population regarding appendicitis [10]. Interestingly, healthcare professionals emerged as the primary source of information, underscoring their pivotal role in disseminating accurate information and addressing misconceptions about appendicitis.

Furthermore, there were indications that prior knowledge may influence respondents' choice of information sources and their perception of the pain associated with appendicitis. This underscores the importance of considering individuals' prior knowledge and its potential effects when managing appendicitis cases and disseminating relevant information.

This study provided insights into respondents' knowledge about appendicitis symptoms and treatments. While the majority of participants were aware of common symptoms like nausea and vomiting, a significant proportion responded with "I don't know." This finding is in line with previous research that has identified gaps in public awareness regarding appendicitis symptoms [11]. The recognition of surgery and antibiotics as common treatment options aligns with established medical practices [12]. About 45% of the respondents mentioned that in the case of acute appendicitis, the use of antibiotics is a better alternative to appendectomy; this supports the 2021 publication of Javanmard-Emamghissi et al., which mentioned that uncomplicated acute appendicitis can be managed by non-operative (antibiotic) treatment [13]. However, the mention of alternative treatments, such as herbs and garlic, indicates the influence of cultural beliefs and practices on individuals' perceptions of treatment options.

Several recent studies [14,15] have explored the potential for non-operative management of acute appendicitis, challenging the traditional approach of immediate surgical intervention. These studies have reported a notable success rate, with some suggesting that uncomplicated cases of acute appendicitis can be managed without surgery. This approach typically involves the administration of antibiotics to control the infection and inflammation of the appendix. While the exact criteria for selecting patients suitable for non-operative treatment may vary, it is essential to note that this alternative approach is gaining recognition in the medical community.

Additionally, while assessing the relationship between gender and respondents' opinions and experiences, there were slight variations in responses between males and females regarding surgery as the best treatment and the perceived risks associated with surgical removal of the appendix suggest the presence of gender-related differences in attitudes and perceptions. These findings align with previous studies that have identified gender-based variations in healthcare-seeking behavior and treatment preferences [16].

Despite the valuable insights provided by the study, it is essential to acknowledge certain limitations. The research was conducted within a specific geographic area, which may limit the generalizability of the findings to other populations. Additionally, the study's reliance on self-reported responses might introduce potential biases and inaccuracies in the data.

## Conclusions

This study has illuminated the prevalent misconceptions and knowledge gaps surrounding appendicitis among residents of the Aseer region in Saudi Arabia. The findings reveal a notable lack of understanding among a significant portion of the population, across various age groups and educational backgrounds. Importantly, the study identifies gender-related variations in perceptions and knowledge regarding appendicitis. While both males and females exhibited limited understanding in some areas, there were noticeable differences in their opinions and experiences related to appendicitis, indicating the presence of gender-related disparities. These findings underscore the urgency of targeted educational interventions and healthcare communication strategies to address these specific gender-based variations in knowledge and attitudes.
